# Rare Heterogeneous Adverse Events Associated with mRNA-Based COVID-19 Vaccines: A Systematic Review

**DOI:** 10.3390/medicines9080043

**Published:** 2022-08-11

**Authors:** Rana I. Oueijan, Olivia R. Hill, Peter D. Ahiawodzi, Pius S. Fasinu, Dorothea K. Thompson

**Affiliations:** 1 School of Pharmacy, College of Pharmacy and Health Sciences, Campbell University, Buies Creek, NC 27501, USA; 2 Department of Public Health, College of Pharmacy and Health Sciences, Campbell University, Buies Creek, NC 27501, USA; 3 Department of Pharmacology & Toxicology, University of Alabama at Birmingham, Birmingham, AL 35294, USA; 4 Department of Pharmaceutical and Clinical Sciences, College of Pharmacy and Health Sciences, Campbell University, Buies Creek, NC 27501, USA

**Keywords:** COVID-19 vaccines, mRNA vaccines, adverse events, myocarditis, pericarditis, thrombocytopenia, allergic hypersensitivities, CNS effects, orofacial reactions, dermatological events

## Abstract

**Background:** Since the successful development, approval, and administration of vaccines against SARS-CoV-2, the causative agent of COVID-19, there have been reports in the published literature, passive surveillance systems, and other pharmacovigilance platforms of a broad spectrum of adverse events following COVID-19 vaccination. A comprehensive review of the more serious adverse events associated with the Pfizer-BioNTech and Moderna mRNA vaccines is warranted, given the massive number of vaccine doses administered worldwide and the novel mechanism of action of these mRNA vaccines in the healthcare industry. **Methods:** A systematic review of the literature was conducted to identify relevant studies that have reported mRNA COVID-19 vaccine-related adverse events. **Results:** Serious and severe adverse events following mRNA COVID-19 vaccinations are rare. While a definitive causal relationship was not established in most cases, important adverse events associated with post-vaccination included rare and non-fatal myocarditis and pericarditis in younger vaccine recipients, thrombocytopenia, neurological effects such as seizures and orofacial events, skin reactions, and allergic hypersensitivities. **Conclusions:** As a relatively new set of vaccines already administered to billions of people, COVID-19 mRNA-based vaccines are generally safe and efficacious. Further studies on long-term adverse events and other unpredictable reactions in close proximity to mRNA vaccination are required.

## 1. Introduction

The development of safe and effective vaccines represents a critical public health measure for the control and mitigation of the ongoing novel Coronavirus Disease 2019 (COVID-19), which is caused by a severe acute respiratory syndrome coronavirus 2 (SARS-CoV-2) infection. Three vaccines for COVID-19 prophylaxis were initially made available to the U.S. public under Emergency Use Authorization (EUA) by the U.S. Food and Drug Administration (FDA): two mRNA-based vaccines (the Pfizer-BioNTech BNT162b2 and Moderna mRNA-1273 vaccines) and Johnson & Johnson’s Janssen adenoviral vector-based vaccine. The Pfizer-BioNTech and Moderna vaccines both utilize a lipid nanoparticle-encapsulated mRNA platform that encodes the prefusion stabilized spike (S) protein of SARS-CoV-2 [1,2]. The surface-exposed S protein is a fusion glycoprotein that mediates host cell recognition and entry by binding to the host cell receptor angiotensin-converting enzyme 2 (ACE2) [3,4]. Both mRNA-based COVID-19 vaccines are administered intramuscularly as a two-dose primary series, and booster doses are available. On 23 August 2021, the BNT162b2 vaccine, now marketed under the brand name Comirnaty^®^, received full U.S. FDA approval for individuals aged 16 years and older [5]. The U.S. FDA approved the second mRNA-based COVID-19 vaccine, known as the Moderna vaccine (now Spikevax^®^), on 31 January 2022, for individuals aged 18 years of age and older [6]. Recently, the U.S. FDA expanded EUA for the primary two-dose regimen of both the Pfizer-BioNTech and Moderna vaccines for use in recipients as young as 6 months of age [5,6].

Clinical trials and nationwide vaccination campaigns have demonstrated the effectiveness of the Pfizer-BioNTech and Moderna vaccines in preventing or mitigating symptomatic disease among vaccinated adults [1,2,7]. While the public health benefits of vaccination are clear, COVID-19 vaccines have been associated with rare adverse events in susceptible individuals. Since the rollout of the mRNA vaccines in December 2020, a number of common, but mild, side effects have been reported for both the Pfizer-BioNTech and Moderna vaccines, including fatigue, headache, muscle pain at the injection site, and fever [8]. However, more severe adverse events, like myocarditis and pericarditis, have been infrequently documented following COVID-19 vaccination, especially in younger patients. The U.S. FDA announced its intention to delay a decision on authorizing the Moderna vaccine for adolescents (ages 12–17) so that the agency could review the risk of developing a rare inflammatory heart condition following Moderna vaccination in the pediatric population [9].

The anti-SARS-CoV-2 vaccination campaign can be described as historical in its proportions, with billions of vaccine doses given worldwide and just over 601 million doses administered in the U.S. alone as of 25 July 2022 [10]. Given the massive number of vaccine doses administered and the novel mechanism of action of these mRNA vaccines in the healthcare industry, a comprehensive review of the more serious adverse events associated with the Pfizer-BioNTech and Moderna mRNA vaccines is warranted to make clinicians aware of the potential risks. Therefore, the aim of this study was to conduct a systematic review of the literature and discuss the clinical presentation and patient demographics of severe adverse effects following immunization with the approved mRNA-based COVID-19 vaccines.

## 2. Materials and Methods

### 2.1. Search Strategy

A comprehensive literature search was conducted initially in January 2022 using the PubMed, Scopus, Google Scholar, LitCOVID, and Centers for Disease Control and Prevention’s (CDC’s) Vaccine Adverse Event Reporting System (VAERS) databases. Updated literature searches were conducted weekly until May 20, 2022. The literature review was conducted using the following pre-specified search terms: COVID-19 vaccines AND myocarditis, COVID-19 vaccines AND pericarditis, COVID-19 vaccines AND thrombocytopenia, COVID-19 vaccines AND allergic reactions, COVID-19 vaccines AND CNS effects, COVID-19 vaccines AND orofacial adverse events, COVID-19 vaccines AND dermatological reactions, COVID-19 vaccines AND skin adverse events, and COVID-19 vaccines AND adverse effects.

### 2.2. Inclusion and Exclusion Criteria

**Figure 1:**
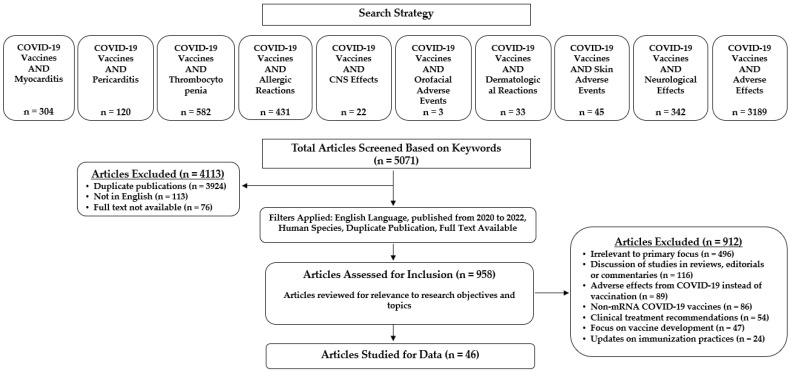
Literature search results and study selection strategy.

Our study selection strategy is shown in Figure 1. Articles retrieved based on a search with pre-specified keywords were initially screened for eligibility by applying the following inclusion criteria: (i) published from 2020 to 2022, (ii) in English, and (iii) full text available. Duplicate publications were excluded. A second screening process excluded full-text articles that were not directly relevant to our research objectives and topics (e.g., articles with a focus on adverse effects of COVID-19 infection, vaccine development, non-mRNA COVID-19 vaccines, or clinical treatment recommendations). Only peer-reviewed studies reporting adverse events following immunization with one of the novel mRNA-based COVID-19 vaccines were included. Two reviewers independently searched the literature, applied screening filters, and evaluated each article’s eligibility for inclusion based on the predefined criteria. Three additional reviewers supervised the study selection process.

### 2.3. Assessment of Study Quality

The quality of selected articles was assessed by two independent and experienced researchers (P.S.F. and D.K.T.) using the Preferred Reporting Items for Systematic Reviews and Meta-Analyses (PRISMA) checklist. Articles were selected if the studies provided supporting laboratory data, histology, imaging findings, or clinical evidence of post-vaccination adverse events.

## 3. Results

Figure 1 schematically depicts the study selection strategy and the search results obtained. Prior to screening, the initial literature search yielded 5071 articles across diverse categories of adverse events that comprised myocarditis, pericarditis, thrombocytopenia, allergic hypersensitivities, central nervous system (CNS) effects, orofacial events, and dermatological reactions. After removing duplicates and applying additional screening filters, the full texts of 958 articles were assessed for eligibility. Forty-six articles met the inclusion criteria and were included in this systematic review (Figure 1).

### 3.1. Myocarditis and Pericarditis

Myocarditis refers to the inflammation of the myocardium, and its etiology can be autoimmune, infectious, or idiopathic in nature [11,12]. Similarly, pericarditis is an inflammatory condition affecting the pericardium, the outer lining surrounding the heart. Acute myocarditis is characterized by an infiltration of immune cells and inflammatory cytokines into the heart, which results in non-ischemic damage to cardiomyocytes [12]. The apical proinflammatory cytokine interleukin (IL)-1 plays a pivotal role in myocardial inflammation [13]. In particular, IL-1α profoundly influences the immune response that leads to myocarditis as it is released from dying myocardium cells [13]. Furthermore, dysregulated autoreactive CD4+ T cells and their cytokines are critical for the autoimmune-related induction of myocarditis in genetically predisposed individuals [14,15,16]. Acute myocarditis can present with nonspecific symptoms such as chest pain or discomfort, dyspnea, dizziness, and arrhythmias. While most cases of myocarditis tend to resolve spontaneously [17], inflammation may progress to a chronic stage in susceptible individuals and eventually result in pathological cardiac remodeling, fibrosis, contractile dysfunction, and life-threatening dilated cardiomyopathy [18].

#### 3.1.1. Incidence of Myocarditis following Non-COVID-19 Vaccinations

Post-vaccination cardiovascular adverse events in the form of myocarditis or pericarditis have been reported previously for the smallpox (vaccinia), conjugate meningococcal C (MENcn-C), and hepatitis B virus (HBV) vaccines, although the incidence was rare [19,20,21]. In one surveillance report of 230,734 U.S. military service members who were vaccinated against smallpox (SPX), 18 cases of myopericarditis occurred 7 to 19 days following the first dose of the SPX vaccine [19]. All 18 individuals were Caucasian males, with a mean age of 26.5 years. A probable causal relationship between vaccination and myocarditis development was supported in this report due to the close temporal clustering (a mean time of 10.5 days to clinical presentation), wide geographic distribution, and lack of data to support other alternative etiologies for myocarditis induction [19]. In the report, the observed development of myocarditis with primary vaccines was 3.6 times higher (95% confidence interval [CI], 3.33–4.11) than the expected rate among unvaccinated individuals. There was no statistical significance found when stratifying the background incidence of myocarditis by age, race, and gender [19]. In a multi-center prospective cohort study, healthy subjects were observed for the onset of new cardiac symptoms and cardiac-specific troponin T elevations after receiving either the SPX vaccine or trivalent influenza (TIV) vaccine [20]. Data gathered in this study showed that symptoms of myocarditis/pericarditis occurred in 10.6% of SPX vaccinees compared to 2.6% of TIV vaccinees within 30 days of immunization. Moreover, the post-SPX incidence rate was 214-times higher than the pre-SPX background population surveillance rate of myocarditis/pericarditis [20].

#### 3.1.2. Characteristics of Patients with Confirmed Myocarditis/Pericarditis Following mRNA COVID-19 Vaccination and Outcomes

Although not reported in the randomized controlled clinical trials for the newly developed mRNA vaccines [1,2], case reports and case series document a rare occurrence of the acute onset of myocarditis/pericarditis symptoms in close temporal proximity to mRNA COVID-19 vaccination. Twenty-eight articles published between May and September of 2021 were reviewed in more depth [22,23,24,25,26,27,28,29,30,31,32,33,34,35,36,37,38,39,40,41,42,43,44,45,46,47,48,49]. Summaries of the included study characteristics are presented in Table 1. Collectively, these case reports/series reported on a total of 90 patients who were diagnosed with myocarditis or pericarditis following vaccination with either the BNT162b2 (Pfizer-BioNTech) or mRNA-1273 (Moderna) vaccine. Frequencies and percentages were used to describe the study population characteristics in terms of the following variables: age category, gender, prior health status, vaccine type, number of doses received, and time to presentation after mRNA vaccination. Common cardiac-specific symptoms included chest pressure and pain (substernal, mid-sternal, or retrosternal), intermittent palpitations, and dyspnea. Patients typically showed biomarker evidence of myocardial injury (elevated troponin levels) and cardiac magnetic resonance (CMR) imaging abnormalities consistent with Lake Louise criteria [50] for confirming suspected cases of myocarditis. Among the case reports/series reviewed, a higher frequency of post-vaccination myocarditis/pericarditis patients were 20 years of age or younger (*n* = 43, 47.8%), and 91% (*n* = 82) were male. The median age was 21 years (range was from 14 to 70 years of age). Time from COVID-19 vaccination to symptom onset was collected for 81 of the total 90 patients. Thirty-six patients, which included 33 males and 3 females, experienced symptoms of acute myocarditis 48 h or earlier from the time of vaccination, whereas 45 patients (42 males and 3 females) manifested symptoms of CMR-confirmed acute myocarditis after 48 h from the time of vaccination. Of note, 75 patients (83%) described in the case reports/series were previously healthy prior to developing myocarditis and had no medical history of cardiac issues. Fifteen patients (16.7%) who developed post-vaccination myocarditis/pericarditis had prior medical conditions that included obesity, hyperlipidemia, obstructive sleep apnea, liver function test (LFT) elevation, asthma, insulin resistance, vitiligo, a prior history of pericarditis, and an episode of atrial fibrillation. A substantially greater proportion of the 90 patients described in the case report/series [22,23,24,25,26,27,28,29,30,31,32,33,34,35,36,37,38,39,40,41,42,43,44,45,46,47,48,49] developed myocarditis/pericarditis after receiving the second dose (*n* = 79, 87.8%) of either the Pfizer-BioNTech or Moderna vaccine. All 90 patients in the reviewed case reports/series either had self-limiting myocarditis/pericarditis or were discharged from the hospital after receiving treatment for their symptoms. There were no reports of fatality among the scrutinized cases.

The case reports and case series reviewed in this section raise more questions than answers. Authors either concluded that a causal relationship between the mRNA vaccine and myocarditis development could not be determined or suggested that myocarditis was a rare adverse effect of the mRNA vaccine. One case series, in particular, stated that although the temporal association between receiving the COVID-19 vaccine and the development of myocarditis strongly suggested a linkage between the two, the authors could not conclude that mRNA vaccination caused the patients’ myocarditis because other etiologies and causes for the cardiac inflammatory event could not be completely ruled out [22]. In another case, an extensive clinical workup was performed to negate other potential causes of myocarditis. After no alternative explanation was identified, the authors concluded that healthcare providers should be cautious with regard to potential myocarditis development post-vaccination in certain clinical situations [26]. Although a causal relationship between mRNA vaccines and myocarditis/pericarditis cannot be definitively concluded, the temporal association should raise a high level of suspicion, especially in cases where all other etiologies have been ruled out [28]. In vaccinated patients with MRI-confirmed myocarditis, recent COVID-19 infection and other possible etiologies were excluded, which raised suspicion of potential vaccine-related, self-limited acute myocarditis [30,33].

#### 3.1.3. Incidence of Myocarditis following mRNA COVID-19 Vaccination

Recently, several retrospective studies conducted in Israel have investigated myocarditis incidence in temporal association with immunization with the mRNA-based Pfizer-BioNTech (BNT162b2) vaccine (see Table 1). Mevorach et al. [51] reviewed data collected from active surveillance initiated by the Israeli Ministry of Health during a nationwide COVID-19 vaccination campaign implemented from December 2020 to the end of May 2021. The authors of this study found that out of the approximately 5.1 million individuals who received the two-dose regimen of the Pfizer-BioNTech vaccine, 136 cases of definite or probable myocarditis had occurred following receipt of the second dose of the vaccine [51]. Consistent with our findings in this review, the susceptible individuals were predominantly male (91%) and under the age of 30 (76%). Myocarditis after the second Pfizer-BioNTech vaccine dose had the highest standardized incidence ratio for male recipients between the ages of 16 and 19 years (13.60 per 100,000 people; 95% CI, 9.30–19.20) [51]. These results were comparable to another study in which post-vaccination myocarditis incidence was estimated using the Israeli Clalit Health Services database. In that study, Witberg et al. [52] found that the highest incidence of myocarditis per 100,000 people who had received at least one dose of the Pfizer-BioNTech vaccine occurred among male patients between the ages of 16 and 29 years old (10.69 cases; 95% CI, 6.93–14.46). In both studies, the clinical presentation of myocarditis following vaccination was generally mild or moderate in severity [47,48]. Finally, Barda et al. [53] evaluated the risk association of the Pfizer-BioNTech vaccine with a broad range of potential adverse events. The authors found vaccination to be most strongly associated with an increased risk of myocarditis (risk ratio, 3.24; 95% CI, 1.55–12.44), but it appeared to be protective against such adverse events as anemia and intracranial hemorrhage [53]. However, the risk of myocarditis was higher after SARS-CoV-2 infection compared to post-vaccination.

### 3.2. Thrombocytopenia

Immune thrombocytopenia (ITP) is defined as a decrease in platelet count, typically below 100 × 10^9^/L (reference range, 150–400 × 10^9^/L) that manifests as variable bleeding symptoms (e.g., petechiae or purpuric skin rashes, gingival bleeding, epistaxis, and easy bruising). The pathophysiology of primary ITP, an acquired immune disorder, is attributed to immune-mediated destruction of platelets, involving antiplatelet antibodies and T cells, and impaired megakaryocytopoiesis [69]. Secondary ITP is associated with other underlying disorders, such as autoimmune disease, immune dysregulation, and certain infections, including COVID-19 [69,70]. The reported annual incidence estimates for acute ITP are approximately 3.3 per 100,000 adults and between 1.9 and 6.4 per 100,000 children [71]. Previously, ITP has been reported to the VAERS passive surveillance system as a rare adverse event following such routine vaccinations as measles-mumps-rubella (MMR), *Haemophilus influenzae* type B, hepatitis B virus (HBV), pneumococcus, human papilloma virus (HPV), varicella-zoster virus, diphtheria-tetanus-acellular pertussis, and polio [72]. It is unclear whether a causal relationship exists between these vaccines and the development of ITP. The cause of vaccine-related thrombocytopenia is thought to be immune-related because antibodies are detected on platelets in the majority of cases [73].

Cases of new-onset ITP occurring post-immunization with the Pfizer-BioNTech and Moderna mRNA vaccines have been reported (Table 1), attracting public attention. In one published case report, a 22-year-old, otherwise healthy, male patient developed purpuric lesions (petechiae) and gum bleeding on day three post-vaccination with the Pfizer-BioNTech BNT162b2 vaccine [54]. Upon presentation, laboratory tests revealed that the patient was in severe thrombocytopenia with a platelet count of 2 × 10^9^/L. Two months prior to the COVID-19 mRNA vaccination, the patient’s routine lab work indicated a platelet count of 145 × 10^9^/L. The patient tested negative for COVID-19, HIV, hepatitis B and C viruses, and Epstein-Barr virus (EBV). By day six, the patient’s platelet count increased to 28 × 10^9^/L and, due to the exclusion of any alternative etiologies, the patient was diagnosed with immune thrombocytopenia [54]. The patient’s platelet count recovered to reference levels by day eleven post-vaccination, and a follow-up assessment indicated that the patient remained healthy without evidence of autoimmune disease.

Other studies have examined multiple cases of symptomatic ITP following vaccination in order to provide insight into the possible relationship, if any, between mRNA COVID-19 vaccines and depressed platelet counts. These case series also have important implications for therapeutic management and surveillance. In one such study, Lee et al. [55] analyzed twenty case reports of patients with thrombocytopenia following vaccination. Only patients who presented with symptoms of secondary immune thrombocytopenia within the first two weeks following vaccination were included in this study. Of the twenty patients, 17 did not have pre-existing thrombocytopenia, suggesting that these cases may have been secondary ITP that developed following vaccination [55]. Nine patients received the Pfizer-BioNTech vaccine, while eleven received the Moderna vaccine. The median age was 41 years (ranging from 22 to 73) and 11 were female. All 20 patients were hospitalized, presenting with petechiae, bruising, and gingival bleeding as well as platelet counts that varied from 1–36 × 10^9^/L, with the majority of patients having counts at or below 10 × 10^9^/L [55]. Several patients had a previous history of thrombocytopenia, and three other patients had autoimmune conditions including hypothyroidism, Crohn’s disease, or detectable anti-thyroglobulin antibodies. Treatment for thrombocytopenia was defined for 15 of the cases, which included intravenous immunoglobulin (IVIG), platelet transfusion, rituximab, romiplostim, vincristine, or aminocaproic acid. Outcomes were reported for 16 of the 20 patients. Of those, 14 experienced improvements in platelet count with treatment, one patient did not experience improvement, and one patient passed away due to cerebral hemorrhage [55]. The authors of the study concluded that they cannot exclude the possibility of the mRNA vaccines potentially provoking new-onset ITP, but it would be very rare [55]. Presently, it is not possible to distinguish between vaccine-induced ITP and coincidental ITP that develops in close proximity following mRNA vaccination. Lee et al. [55] recommend a baseline platelet count in patients with pre-existing thrombocytopenia before receiving either the Pfizer-BioNTech or Moderna vaccine and a follow-up platelet count after vaccination. This case series also showed possible treatment options for patients with potential secondary ITP. Most patients responded favorably to treatment with corticosteroids or IVIG, which points to an immune-mediated mechanism characterizing post-vaccination ITP. In general, patients showed little improvement with platelet transfusion, and no response was observed in two patients administered rituximab [55].

In another case series, Welsh et al. [56] analyzed adverse events data reported to VAERS and found 15 reports of thrombocytopenia associated with the Pfizer-BioNTech vaccine and 13 cases associated with the Moderna vaccine out of 18,841,309 and 16,260,102 doses of vaccines distributed, respectively, in the U.S. as of 4 February 2021. This corresponded to a reporting rate of thrombocytopenia of 0.80 per million doses for both vaccines [56]. Two cases of thrombocytopenia occurred after the second vaccine dose, while the remaining cases occurred after the first dose, or the dose number was not reported. Onset of thrombocytopenia symptoms generally occurred 1 to 23 days after vaccination, with a median onset to presentation of 5.5 days. The age of patients ranged from 22 to 82 years, with the median age being 48.5 years. Not all patients were previously healthy, and many individuals had comorbidities such as type I diabetes, Crohn’s disease, and Hashimoto’s thyroiditis. Three patients had a prior history of ITP. Treatment included prednisone, platelet transfusions, IVIG, and rituximab, among others, with most cases resolving and patients being discharged [56]. Two cases resulted in death, which was attributed to intracranial hemorrhaging secondary to ITP in one patient and acute myocardial infarction in another. Based on the very low number of thrombocytopenia cases reported to VAERS compared to the incidence rate of ITP estimated among unvaccinated adults, the authors of this study concluded that a safety concern attributed to the COVID-19 mRNA vaccines was not warranted at this time [56].

In rare instances, post-vaccination ITP events were refractory to first-line treatment. One case report described a 74-year-old male patient who developed severe, refractory ITP one day after receiving the first dose of the Moderna COVID-19 vaccine in January 2021 [57]. Two months before immunization, a platelet count of 224 × 10^9^/L was recorded, but upon presentation post-vaccination, his platelet count had declined to 10 × 10^9^/L. The patient presented with uncontrollable nose bleeds and diffuse cutaneous purpura consistent with thrombocytopenia within hours of vaccination, and on post-vaccination day 13, developed generalized muscle weakness and encephalopathy, although these conditions were considered to be unrelated to the ITP [57]. A previous medical history of hypertension, hyperlipidemia, gout, and nonischemic cardiomyopathy was reported for this patient. The hospitalized patient was treated with high-dose dexamethasone (40 mg/day, post-vaccination days 1–6), five daily doses of IVIG (400 mg/kg/day), three daily platelet transfusions, and two weekly doses of rituximab (375 mg/m^2^/dose). The improvement in platelet count was not significant, marking the patient’s case of thrombocytopenia as refractory to first-line treatment. On post-vaccination day 10, the patient received four days of eltrombopag (50 mg/day), a thrombopoietin receptor agonist (TPO-RA). There was no improvement in the patient’s platelet count, and his case of thrombocytopenia was designated as refractory to second-line treatment. On post-vaccination day 15, the patient received treatment with plasma exchange, high-dose methylprednisolone (1 mg/kg/day), and TPO-RA romiplostim (5 mcg/kg). The patient’s platelet counts began to trend upwards within five days of this treatment and returned to normal range by post-vaccination day 25. The authors of this report stated that it is uncertain whether refractory ITP secondary to vaccination with a mRNA COVID-19 vaccine will be a rare adverse event [57]. Nonetheless, this uncertainty should not limit the use of mRNA vaccination as a public health measure to prevent or mitigate COVID-19 infection. More research is needed to understand the pathogenesis, epidemiology, clinical profiles, and management of ITP associated with these novel mRNA vaccines [57].

### 3.3. Allergic Hypersensitivities

The majority of common adverse reactions attributed to vaccinations are not immunologically mediated and occur as a result of the pharmacology of the vaccine, its excipients, or inactive ingredients in the formulation [74]. Non-immunologically mediated reactions typically include toxic effects and medication interactions. By contrast, anaphylactic allergic reactions to vaccinations, although extremely rare, are typically triggered by an IgE-mediated mechanism that involves prior exposure to an allergen in a genetically predisposed individual and the production of allergen-specific IgE antibodies [75,76]. Immunologically mediated reactions can also include T cells and other immunologic mechanisms. An anaphylactic reaction (or immediate-type hypersensitivity) to a vaccine generally occurs within minutes to an hour or more after allergen exposure and constitutes a multisystem, potentially life-threatening event due to the widespread release of histamine and other vasoactive mediators.

A recent systematic review and meta-analysis study estimated the incidence rates of anaphylactic and non-anaphylactic reactions reported following administration of either the Pfizer-BioNTech or Moderna mRNA COVID-19 vaccine in an adult population [77]. Various electronic databases were searched and included 26 relevant articles published during the period of December 2020 to May 2021. Most of the 26 included studies reported anaphylactic and nonanaphylactic reactions after the first vaccine dose (*n* = 14), while others described reactions following the second vaccine dose (*n* = 8), or the dose number was not reported. The overall estimated pooled prevalence for anaphylactic events to both mRNA vaccines was 5.58 (95% CI 3.04–8.12, *p* = 0.00) per million doses, while the overall pooled prevalence estimate was significantly higher for nonanaphylactic reactions to both vaccines at 89.53 (95% CI 11.87–190.94, *p* = 0.00) per million doses [77]. A higher incidence of anaphylaxis was associated with the Pfizer-BioNTech vaccine (9.31 per million doses administered, 95% CI 4.23–14.40) compared to the Moderna vaccine (3.42 per million doses administered, 95% CI 1.42–5.41). However, Moderna vaccination resulted in higher nonanaphylactic reactions (99.01 per million doses administered, 95% CI 49.77–247.80) compared to Pfizer-BioNTech (75.27 per million doses administered, 95% CI 48.28–198.82) [77].

In cases of diagnosed anaphylaxis, common presentation symptoms included pruritic hives, throat closure, angioedema, wheezing, nausea and vomiting, tachycardia, hypotension, dyspnea, and tongue swelling. Nonanaphylactic adverse events following mRNA COVID-19 vaccination predominantly manifested as cutaneous reactions and delayed large local reactions such as injection site swelling/pain, erythema, rash, and urticaria. Female gender and a previous history of atopy were the most frequently identified risk factors for anaphylactic and nonanaphylactic reactions to the SARS-CoV-2 mRNA vaccines. Patients with a known history of anaphylaxis, dermatologic comorbidities (such as atopic or contact dermatitis), asthma, or allergic rhinitis were more susceptible to allergies associated with these vaccines [58,59]. It has been hypothesized that the polyethylene glycol (PEG)-conjugated lipid derivative in the formulation of the SARS-CoV-2 mRNA vaccines may be an antigen for anaphylaxis and nonanaphylactic reactions [78]. The female predominance in reported cases of vaccine-associated anaphylactic and nonanaphylactic reactions may be the result of a higher frequency of sensitization to PEG through the use of such PEG-containing products as cosmetics [79]. Despite the higher risk to females, Alhumaid et al. [77] concluded that the results of their systematic review and meta-analysis should not dissuade individuals from receiving the mRNA COVID-19 vaccines. The prevalence of mRNA vaccine-associated anaphylaxis is very low, and although nonanapylactic reactions occur at a higher rate, the cutaneous manifestations are largely self-limiting [77]. 

### 3.4. CNS and Orofacial Events

CNS and orofacial adverse reactions following mRNA COVID-19 vaccination have been reported in observational cohort studies, case reports, and case series. Documented neurological adverse events include CNS syndromes, cerebrovascular disorders, and peripheral nervous system disorders. Bell’s palsy, a rare idiopathic peripheral facial paralysis, has been reported as an adverse reaction to the novel anti-SARS-CoV-2 mRNA vaccines, as well as cerebral venous thrombosis, and acute transverse myelitis [80]. Besides Bell’s palsy, other vaccine-associated orofacial adverse effects include facial swelling and swelling of the lips. A literature search from January 2022 through May 2022 identified six studies that met our inclusion criteria (see Table 1). The articles included two prospective observational cohort studies [60,61], one case report [62], two case series [63,64], and one survey-based study [65] and are discussed in more depth here. These studies focused on neurological disorders and complications in temporal proximity to vaccination with the BNT162b2 (Pfizer-BioNTech) and mRNA-1273 (Moderna) vaccines.

A multi-center prospective observational cohort study conducted in Singapore centered around seven public acute hospitals [60]. Patients who presented with neurologic complaints and had at least one mRNA vaccine dose within the last 6 weeks were classified into CNS syndromes, cerebrovascular disorders, autonomic nervous system disorders, peripheral nervous system disorders, and immunization stress-related responses. This study covered an observational period from 30 December 2020, to 20 April 2021. It is important to note that the Pfizer-BioNTech vaccine was approved in Singapore on 30 December 2020, while the Moderna vaccine was not approved until 12 March 2021. A total of 1,398,074 individuals in Singapore received at least one dose of a mRNA COVID-19 vaccine during the observational period, with the majority receiving the Pfizer-BioNTech vaccine (86.7%) compared to the Moderna vaccine (13.3%). Of the total number of vaccinated individuals, 457 patients were referred for neurological complaints within 6 weeks of immunization [60].

The most common post-vaccination CNS syndrome was seizures, with 33 patients (median age of 63 years) experiencing this adverse event [60]. Seventeen (51.5%) of these patients were males. Thirty-one patients received the Pfizer-BioNTech vaccine, while two received the Moderna vaccine. Only 17 seizures constituted first-onset seizures, with four being characterized as first-onset unprovoked. The remaining patients reported a pre-existing history of epilepsy. Other CNS syndromes included encephalopathy (*n* = 4) and demyelinating diseases (*n* = 4). Acute ischemic stroke (AIS) was the most common cerebrovascular disorder seen in this observational cohort study [60]. Of the 457 hospitalized patients included, 243 experienced neuroimaging-confirmed AIS, with all of these patients having at least one underlying cerebrovascular risk factor and 41 undergoing revascularization treatments. Eleven patients were 50 years of age or younger. Of the 243 patients diagnosed with AIS, 234 were immunized with the Pfizer-BioNTech vaccine and 12 with the Moderna vaccine. The majority of these patients (*n* = 144) had a good functional outcome upon hospital discharge, classified by a 0–2 on the modified Rankin Scale. Two of the 457 patients experienced cerebral venous thrombosis (CVT) [60]; one patient remained neurologically debilitated after treatment for 6 weeks, while the other recovered and was discharged. Of the 59 patients with peripheral nervous system disorders, the most common presentation was Bell’s palsy, which occurred in 11 patients (5 of whom were males) with a median age of 66 years [60]. All 11 patients received the Pfizer-BioNTech vaccine.

An immunization stress-related response was observed in 39 patients who received the Pfizer-BioNTech vaccine [60]. The median age for these patients was 51 years, and 16 were males. Immunization stress-related responses included sensory complaints, dizziness, headaches, focal twitching, unsteadiness, abnormal movement/twitching, and visual blurring. The authors stated that their observational study does not establish a causal relationship between the reported neurological complications and recent mRNA vaccination [60]. Furthermore, no neurological morbidity was found. The authors therefore concluded that the benefits of mRNA COVID-19 vaccination exceed any concerns about neurological adverse effects.

Another prospective observational cohort study was conducted in Mexico using data collected from 704,003 first-dose recipients of the Pfizer-BioNTech mRNA vaccine [61]. In this nationwide study, adverse events following immunization were categorized as neurologic if they included at least one of the following: headache, motor/sensory symptoms, focalizing signs, and altered mental status. Guillain-Barré syndrome (GBS), acute transverse myelitis, seizures, and acute palsy or paralysis were considered to be serious neurologic adverse events. During the study’s observational period (from 24 December 2020, to 12 February 2021), 4258 neurological adverse events following Pfizer mRNA vaccination were reported. Of these cases, 1016 were males, and the median age was 36 years. Non-serious neurologic adverse events post-vaccination had an overall incidence rate of 600.7 cases per 100,000 administered doses, with headache (62.2%), transient sensory symptoms (3.5%), and weakness (1%) representing the most frequent complaints [61]. Among the 4258 neurological adverse events reported, 17 were classified as serious: seven cases were seizures with an incidence of 0.99/100,000 doses, three corresponded to GBS (0.43/100,000 doses), and two corresponded to acute transverse myelitis (0.28/100,000 doses). At the conclusion of this observational study, 16 of the 17 serious cases of neurological adverse events had been discharged from the hospital, and there were no observed deaths due to neurological adverse events following mRNA vaccination. Interestingly, this study showed that a disproportionate percentage of females experienced neurological adverse events following immunization compared to the percentage of females who were first-dose vaccine recipients. Only 26.8% of the Pfizer mRNA vaccine was administered to females in the study, but 76.3% of the neurologic adverse events reported were experienced by women [61]. The authors stated that this disproportionality has been observed with other vaccines and is likely multifactorial in etiology [61]. Overall, the study by García-Grimshaw et al. [61] showed that the Pfizer-BioNTech vaccine is predominantly safe and effective, and that concerns about adverse events should not diminish the use of vaccination for reducing COVID-19 severity and mortality.

Occurrences of demyelinating disorders, such as acute transverse myelitis (ATM), have been reported following HBV, tetanus, and influenza vaccination [81], although no association between ATM and prior immunization was found [82]. ATM was recently described as a rare complication after mRNA COVID-19 vaccination [63]. The first case was an 81-year-old Korean male who presented with bilateral hand weakness and numbness in the fingers just 3 days after receiving his second dose of the Pfizer-BioNTech vaccine. The patient’s prior medical history was significant for hypertension and diabetes mellitus but had no neurological or sensory symptoms. A spinal MRI revealed multifocal nodular enhancement and a signal increase on T2-weighted images from the C1 to C3 vertebrae. The second case, a 23-year-old Korean female with no significant medical history, presented with a tingling sensation and weakness in the legs three weeks after the first dose of the Pfizer mRNA vaccine [63]. The patient became unable to walk, and a spinal MRI revealed a lesion with high signal intensity at the anterior part of the conus medullaris on T2-weighted images. Both patients were treated with intravenous methylprednisolone. One month following the end of treatment, the male patient still experienced limitations in finger movements. Three weeks after treatment, the leg weakness in the female patient improved and she could walk with unilateral assistance. Causation between these patients’ neurological symptoms and mRNA COVID-19 vaccination could not be established [63].

Besides ATM, other CNS demyelinating events, like multiple sclerosis (MS), neuromyelitis optica spectrum disorder, and myelin oligodendrocyte glycoprotein antibody disease, have occurred after administration of all types of approved COVID-19 vaccines, including the mRNA versions [83]. In a previous systematic review, the authors found a female predominance (68.8%) among reported post-COVID vaccination cases of CNS demyelination reported across 12 countries, including the U.S. [83]. The median patient age was 44 years, and the median time from COVID vaccination to symptom onset was 9 days. For 71.8% of patients, the beginning of neurological symptoms occurred after the first dose of the COVID-19 vaccine, and 53.1% of patients had been previously diagnosed with an immune-related condition such as MS and had experienced recurrent neurological symptoms. Of the reported presentations, the most common were transverse myelitis and MS. A favorable outcome was observed in the majority of cases after treatment. Ismail and Salama [83] concluded that the incidence of post-vaccination CNS demyelination appears to be low when compared to demyelination following COVID-19 infection. The authors also emphasized the need for long-term surveillance of CNS demyelination as a potential adverse event of COVID-19 vaccines in order to assess causality and ensure vaccine safety.

Another rare neurological event was documented in a 42-year-old Japanese female who was diagnosed with aseptic meningitis one week after her first intramuscular dose of the Pfizer-BioNTech vaccine [62]. The patient initially presented with a severe headache and high fever (38 °C); she was positive for jolt accentuation with headache and nausea upon hospital admission, but was negative for neck stiffness, Kernig sign, and Brudzinski sign. The patient’s C-reactive protein (CRP) level was increased to 9.85 mg/dL (normal range, 0–0.3 mg/dL), and a cerebrospinal fluid (CSF) analysis revealed pleocytosis. The patient was treated with intravenous methylprednisolone for five days, after which her symptoms improved and her serum CRP and pleocytosis resolved. These results suggest a rare type of immune-mediated response as the cause of the patient’s aseptic meningitis [62]. Molecular mimicry induced by the vaccine-produced SARS-CoV-2 spike protein may have caused autoimmune meningitis in this patient [62], although no evidence currently exists for such a mechanism.

Although uncommon, clinicians should be aware that orofacial adverse events have been reported in individuals who received a mRNA COVID-19 vaccine. Bell’s palsy, facial swelling, and tongue/lip swelling are some of the more common orofacial adverse events described. In two large Phase 3 clinical trials, Bell’s palsy was reported [1,2], but the incidence was not significantly different from the frequency of Bell’s palsy found in the general population [84]. A survey-based study was conducted in March 2021 to investigate oral and facial manifestations of post-COVID-19 vaccination [65]. Potential participants included individuals who were medical staff (e.g., physicians, nurses, and dentists), had access to COVID-19 vaccines, and had been immunized with at least one vaccine dose. A total of 700 individuals received the survey across Poland, Italy, and other European Union countries. A total of 223 people responded (74.4% were female). The Pfizer-BioNTech vaccine was administered in 217 cases, with the Moderna vaccine administered in only one case. Of the 223 respondents, 182 had received both mRNA vaccine doses. The survey questions investigated changes in facial sensitivity, paresis, paralysis, and aesthetics. Oral symptoms, such as burning, xerostomia, tongue depapillation, and pain, were also queried. Overall, changes in facial sensitivity were reported in 2.7% and 3.1% of respondents who had received one or two vaccine doses, respectively [65]. A burning sensation was the most common oral adverse effect, with a 2.7% and 3.4% increase in first- and second-dose vaccine recipients, respectively. No causation or significant correlation was found between COVID-19 vaccines and orofacial adverse effects in this study [65].

In a recent case series, oral adverse reactions following COVID-19 vaccination have been described as quite painful. Nine Korean patients complained of oral pain and discomfort less than one month after the time they received a COVID-19 vaccine; four of these patients were immunized with the Pfizer-BioNTech mRNA vaccine [64]. Patients were older, with a mean age of 73.3 years, and experienced a range of oral mucositis, ulceration, and neuropathic pain. For those who received the Pfizer vaccine, the time from vaccination to symptom onset was approximately 2 days. As in the case of other post-vaccination adverse events, patients responded well to treatment.

### 3.5. Dermatological Reactions

Adverse dermatological events, such as rashes, injection-site reactions, and even alopecia areata, have been documented as possible side effects of mRNA COVID-19 vaccination. A registry-based study was conducted to characterize the morphology and timing of cutaneous manifestations following administration of the novel mRNA vaccines ([66] (see Table 1)). The international registry represented a collaborative endeavor between the American Academy of Dermatology and the International League of Dermatological Societies; case entry was restricted to healthcare workers only. The vaccine arm of the registry collects information about the timing of the vaccine doses, morphology, duration of the dermatological reaction, and treatment. From 24 December 2020, to February 14, 2021, this registry recorded 414 unique patient cases of cutaneous reactions to the Moderna (83%) and Pfizer-BioNTech (17%) vaccines [66]. A wide spectrum of post-vaccination dermatological events was reported, from common injection-site reactions to urticaria, morbilliform eruptions, and more atypical manifestations such as erythromelalgia and pityriasis-rosea-like eruptions. Reported cases were predominantly female (90%), white (78%), and from the U.S. (98%), with a median age of 44 years. Of the 414 patient cases evaluated, data on both mRNA vaccine doses was available for only 180 patients. Of those 180, 21% of the cutaneous reactions occurred after the first vaccine dose, 63% after the second dose only, and 16% of the reactions were reported with both doses [66]. The median time from first vaccination to symptom onset was 7 days, while the median time from second vaccination to symptom onset was 1 day. In addition, a delayed large local arm reaction (DLLR)—characterized by a patch of erythema, induration, and tenderness at the injection site—occurred more frequently after receipt of the Moderna vaccine (94%), with a median time to presentation onset of 7 days after the first dose. DLLRs developed more rapidly following the second Moderna vaccine dose (median time of 2 days). The etiology of DLLRs is unknown, but the pathology is consistent with delayed-type hypersensitivity [66]. Results from this study demonstrated that cutaneous side effects of COVID-19 vaccination are usually self-limited without any therapeutic intervention, and the risk of such adverse events does not outweigh the health benefits of immunization against SARS-CoV-2 [66].

Other studies also found that cutaneous adverse reactions following mRNA COVID-19 vaccination were most commonly reported in females and middle-aged individuals [85]. Injection-site redness was the most frequently chronicled dermatological reaction to all COVID-19 vaccine types, followed by itchiness, rash, morbilliform eruptions, and pityriasis rosea. Consistent with other published reports, no long-term cutaneous sequelae generally remained in patients, and most dermatological reactions were self-limiting [85].

With more serious cutaneous manifestations, therapeutic intervention has improved outcomes. In one case report, treatment with clobetasol resolved a male patient’s pruritic rash, which presented as clusters of erythematous papules and nodules and developed about one week after he received his first mRNA vaccine dose [67]. In two other cases of cutaneous eruptions, a female patient in her 20s with a prior medical history of alopecia areata developed a large, itchy, red, and scaly plaque at the immunization site two days after her first dose of the Pfizer-BioNTech mRNA vaccine [68]. Subsequently, she experienced lesions on her torso. After receiving the second dose, her symptoms worsened with increased itching and the number of lesions. The patient was diagnosed with a pityriasis rosea-like eruption and started on topical corticosteroids. A second patient in his 40s presented with a red, scaly plaque on his lateral left axilla 3 weeks after receiving his second dose of the Pfizer mRNA vaccine [68]. He was diagnosed with a pityriasis rosea-like eruption and was given doxycycline and bilastine. The symptoms of both patients resolved following treatment. Although further research is needed to determine if a causal relationship exists, healthcare providers should be cognizant of the variety of cutaneous manifestations that have been reported after mRNA COVID-19 vaccination. For individuals with a history of local injection site reactions or other vaccine-related cutaneous side effects, clinicians should consider pre-vaccination counseling that provides guidance on the use of topical medications to alleviate possible symptoms. Such actions may help to reduce vaccine safety concerns and hesitancy.

## 4. Discussion

The FDA-approved BNT162b2 (Pfizer-BioNTech) and mRNA-1273 (Moderna) vaccines have gained global widespread use in an unprecedented effort to control and mitigate the disease severity of SARS-CoV-2 infection. Both vaccines utilize a novel platform, mRNA, which has never been used previously in a licensed vaccine for humans. Evidence supporting the safety of COVID-19 mRNA vaccines is currently based on Phase 1–3 randomized controlled trials and vaccine safety surveillance systems. A recent meta-analysis study by Wu et al. [86] found that authorized COVID-19 vaccines, including the novel mRNA vaccines, have an acceptable short-term safety profile based on available data. While the protective benefits of COVID-19 vaccination are indisputable, it is important for clinicians to be aware of the heterogeneity of post-vaccination adverse events that have been reported since the initial administration of the mRNA vaccines in December 2020. Such knowledge is key to providing proper guidance to patients regarding vaccination and potential risks. In this review, we focused on providing a comprehensive summary of the more serious, albeit rare, adverse events reported following mRNA COVID-19 vaccination.

Myocarditis and pericarditis are polymorphic inflammatory processes with an indeterminate etiology and variable clinical presentation. New onset myocarditis has been reported as a rare complication of vaccination with the smallpox vaccine [19,20] and most recently, with the mRNA COVID-19 vaccines [87]. The 28 case reports and case series reviewed here were used to extract demographic and clinical information on 90 patients diagnosed with myocarditis/pericarditis following vaccination with either the Pfizer-BioNTech or Moderna mRNA vaccine. The risk of post-mRNA vaccination myocarditis was highest among adolescent and young adult males with no previous comorbidities or history of cardiac issues. This observation is consistent with reports published elsewhere [51,52,88]. The reason for male predominance in post-mRNA vaccination myocarditis cases is currently unclear but may be connected to sex hormone differences in immune response to viral vaccines and cardiac disorders [87]. However, underdiagnosis of myocarditis in females may be a contributing factor [51,87,89]. While a definitive causal relationship between mRNA vaccines and acute-onset myocarditis has not been established, several possible immunological mechanisms for mRNA-related myocarditis have been suggested. One potential mechanism is molecular mimicry between the mRNA vaccine-encoded spike glycoprotein of SARS-CoV-2 and self-antigens, resulting in the formation of antibodies that cross-react with human α-myosin proteins and induce inflammation [90]. Alternative hypotheses include the generation of humoral immune responses to cardiomyocytes bound by the mRNA vaccine-encoded viral spike protein via the ACE2 receptor and complement activation by immune complexes comprised of antibodies directed against anti-spike antibodies [89]. Individuals susceptible to myocarditis after vaccination may be genetically predisposed to autoantibody formation [89]. For the cases reviewed in this study, the clinical course of post-vaccination myocarditis was typically transient and mild, followed by complete resolution of symptoms. Nonetheless, given the expansion of mRNA vaccine authorization for use in very young individuals, healthcare providers should be cautious of the potential risk of such adverse events in certain patient populations. Mechanistic and follow-up studies are needed to ascertain any long-term impacts of myocarditis following mRNA COVID-19 vaccination.

Recently, the ChAdOx1 nCoV-19 (Oxford-AstraZeneca) and Ad26.COV2.S (Johnson & Johnson) vaccines, both COVID-19 adenoviral vector-based vaccines, raised public health concern due to a temporal association with cases of thrombosis with thrombocytopenia syndrome (TTS) [91,92,93,94]. While not completely understood, the mechanism underlying platelet destruction is likely immune-mediated, involving the production of antibodies that act against platelet antigens [95]. Rare occurrences of ITP have also been reported following exposure to the Pfizer-BioNTech and Moderna mRNA vaccines, although not recorded in the clinical trials for these vaccines. The present review found that most patients presenting with ITP developed severe thrombocytopenia after the first mRNA vaccine dose and had various bleeding symptoms (e.g., gingival bleeding, epistaxis, petechiae, or diffuse bruising). Patients generally responded well to immunosuppressive therapy, with platelet counts recovering to reference levels. In some cases, patients had a history of ITP or other autoimmune disorders like type I diabetes or Crohn’s disease. None of the case reports/series reviewed here provided insight into whether patients presented with vaccine-induced secondary ITP or coincidental primary ITP that developed after vaccination. A recent study by Ostrowski et al. [96] demonstrated that both the AstraZeneca (AZ) and mRNA vaccines for COVID-19 amplified inflammation and platelet activation in certain vaccinated individuals, but the AZ vaccine induced higher increases in inflammatory mediators (e.g., TNF-α, IL-1β, and IL-8) and platelet activation markers compared to the mRNA vaccines. Thrombin generation was also higher following AZ vaccination compared to mRNA vaccination [96]. The specific vaccine triggers of inflammation, platelet activation, and thrombin generation are currently unknown. Given the rare occurrence of vaccine-associated ITP, however, the potential risk of this adverse event should not limit the administration of the mRNA COVID-19 vaccines. Clinicians should assess platelet counts of individuals who report abnormal bleeding or bruising following mRNA vaccination and pursue aggressive treatment if necessary.

Reports of adverse allergic reactions to mRNA COVID-19 vaccines have been submitted to VAERS since the initiation of the mass vaccination campaign in December 2020. Using suspected anaphylactic reactions submitted to VAERS, the CDC estimated the rates of anaphylactic cases to be 11.1 per million doses of the Pfizer-BioNTech vaccine (from 14 December to 23 December 2020) and 2.5 per million doses of the Moderna vaccine (from 21 December 2020, to 10 January 2021) [97,98]. Consistent with the articles reviewed in the present study, the early CDC report found a strong female predominance in anaphylactic reactions to both COVID-19 mRNA vaccines [97,98]. The majority of patients were predisposed to allergic adverse events because they had a history of allergic reactions to unrelated medication or other triggers (e.g., food, insect venom), and some had experienced previous anaphylactic reactions to other vaccines. Vaccine-associated anaphylaxis and other hypersensitivities may be caused by IgE-mediated reactions to excipients (preservatives, stabilizers, or adjuvants) in the vaccine formulation that act as allergenic triggers in susceptible individuals and, to a lesser extent, by the viral antigen itself [99]. PEG in the PEGylated lipid nanoparticle, which stabilizes the SARS-CoV-2 mRNA, comprises both mRNA vaccines, and is a potential allergic sensitizer [100]. While PEG is a novel vaccine excipient, this hydrophilic polymer is used extensively in cosmetics and medicaments, as well as in pharmaceutical and food products. Findings reported by Warren et al. [101] suggest that many documented cases of allergy to mRNA vaccines may be the result of non–IgE-mediated hypersensitivity reactions to PEG. A confirmed severe allergy to PEG would be a contraindication to receiving the mRNA vaccines [99]. 

All types of approved COVID-19 vaccines have been associated with rare adverse neurological events. Cases of CNS demyelination, for example, have been described in close temporal proximity to the administration of COVID-19 vaccines, including the mRNA vaccines [83], although a causal linkage has not been established. As discussed in this review, the clinical presentation was heterogeneous and included first-onset seizures with no pre-existing epilepsy, CNS demyelinating syndromes such as acute transverse myelitis and MS-like episodes, and an extremely rare complication of aseptic meningitis. Bell’s palsy was a more common peripheral nervous system syndrome reported among patients who experienced adverse neurological events following mRNA COVID-19 vaccination. A higher prevalence of CNS demyelinating disorders occurred with the mRNA COVID-19 vaccines (53.1%) compared to the viral vector vaccines (31.2%) as reported in one study [83]. TM and MS-like events, particularly in female patients, were the most common demyelinating presentations reported in the articles reviewed here. The majority of adverse neurological cases reported a favorable patient outcome following therapeutic intervention with high-dose methylprednisolone, plasma exchange, IVIG, or a combination thereof. The exact pathological mechanisms of CNS demyelination and other neurological adverse events following mRNA vaccination are poorly understood. One theory involves molecular mimicry, in which the vaccine antigen structurally resembles a self-antigen (e.g., myelin) and induces the production of undesired, cross-reacting antibodies and/or the activation of autoreactive T cells [61]. While potentially serious, vaccine-related CNS demyelinating occurrences are rare, particularly in comparison to demyelination following COVID-19 infection [83,102].

### Limitations

Some limitations need to be considered when assessing the findings of the current systematic review, which focuses on evolving post-vaccination global phenomena. The available literature reviewed in this study largely comprised case reports and case series, with a few retrospective studies and prospective observational cohort investigations. Therefore, this review may have been subject to underreporting or publication bias. Reports in non-English languages were not accessed, which could be a limitation since mRNA COVID-19 vaccination has been globally promoted and adopted. Additionally, a direct causal relationship between mRNA COVID-19 vaccination and the infrequent adverse events discussed here cannot be inferred. A major limitation in most of the included articles is the inability to establish a causal relationship between the reported adverse events and the mRNA vaccines. The most reliable study design for establishing causality is prospective randomized controlled trials. Since the reviewed studies are mostly case reports/series, they only showed temporal associations at best. While this can serve as a basis for caution in the clinical use of the mRNA vaccines, a stronger level of evidence is still necessary. Another general limitation is the number of actual cases and individuals covered in the published studies. In comparison to the billions of people worldwide who have safely received the mRNA COVID-19 vaccines, the number and spread of reported adverse events are insignificant and cannot be said to be sufficiently representative. 

## 5. Conclusions

The novel Pfizer-BioNTech and Moderna mRNA vaccines continue to serve as effective and critical tools in the healthcare industry’s anti-COVID-19 arsenal for reducing the morbidity and mortality of SARS-CoV-2 infection. Common local and systemic adverse reactions to mRNA COVID-19 vaccination include injection-site pain and tenderness, fatigue, and headache. However, clinicians need to be aware of rare, more serious adverse events that have occurred in close temporal proximity to mRNA vaccine administration—namely, myocarditis, immune thrombocytopenia (ITP), anaphylaxis and other allergic hypersensitivities, CNS and orofacial effects, and dermatological reactions. In this review, a robust male predominance in vaccine-associated myocarditis was observed among reported cases, particularly after the second-dose mRNA vaccine. Patients were predominantly young (≤25 years), male, and previously healthy with no prior history of cardiac disease. Cases indicate a female predominance in anaphylactic reactions and cutaneous adverse events following exposure to both mRNA vaccines. Females with a previous history of allergic reactions also had an elevated risk of mRNA vaccine allergy (both anaphylactic and nonanaphylactic). Neurological adverse events included CNS demyelinating disorders, such as acute transverse myelitis (ATM), and peripheral nervous system disorders (e.g., Bell’s palsy). Most patients presenting with ITP developed severe thrombocytopenia after the first mRNA vaccine dose and had various bleeding symptoms (e.g., gingival bleeding, epistaxis, petechiae, or diffuse bruising). The post-vaccination incidence of these heterogenous adverse events was rare in light of the billions of individuals worldwide who have received at least one dose of a mRNA vaccine, although the reports largely reflect occurrences in the U.S. and other developed countries. Moreover, the adverse conditions were generally self-limiting, and patients responded well to treatment options. Further research and long-term population-level surveillance are needed to assess the possibility of causality and the pathological mechanisms underlying these adverse reactions. As vaccination rates continue to increase in populations, clinicians should be able to rapidly recognize symptoms of more serious vaccine-associated adverse events for prompt assessment and initiation of warranted therapeutic intervention. 

## Figures and Tables

**Table 1:** Summary of included studies of adverse events following mRNA COVID-19 vaccination.

Reference	Study Design	Cases, *n*	Country	Description	Outcome	Major Findings/Conclusions
**Myocarditis and Pericarditis**						
Albert et al., 2021 [22]	Case report	1	USA	A 24-year-old male developed symptoms of myocarditis 4 days after a second dose of Moderna vaccine; time to presentation was 96 h post-vaccination. Fulfillment of Lake Louise criteria confirmed acute myocarditis diagnosis.	Transthoracic ECG and LVEF were within normal limits 4 days post-vaccination; cardiac MRI was consistent with findings of myocarditis 5 days post-vaccination. Case was non-fatal with the patient discharged after management.	Data suggested that myocarditis is a slight risk post-COVID-19 vaccination, but no definite association between the Moderna vaccine and post-immunization myocarditis can be made.
Dickey et al., 2021 [23]	Case series	6	USA	Male patients were diagnosed with myocarditis within 2–4 days post-mRNA vaccination (five received second dose of Pfizer-BioNTech vaccine; one received second dose of Moderna vaccine). Time to presentation was 48–96 h post-vaccination. Myocarditis diagnosis confirmed by CMR imaging.	No patients experienced complications; all were discharged home after management.	Clinical presentation and temporal association, when paired with CMR findings, suggest association between vaccination and myocarditis development. However, no definite conclusion can be made as other etiologies cannot be ruled out. Providers should be wary of potential myocarditis development in recently vaccinated patients.
Marshall et al., 2021 [24]	Case series	7	USA	Acute myocarditis or perimyocarditis in seven male adolescents who were otherwise healthy. All developed symptoms within 4 days after receiving second dose of Pfizer-BioNTech vaccine. Time to presentation was 24–48 h post-vaccination. All patients were given cardiac MRIs, which were diagnostic for myocarditis based on 2018 Lake Louise criteria.	All patients resolved symptoms rapidly.	There is no possibility of establishing a relationship between vaccination and myocarditis development based on the data from this case series.
Rosner et al., 2021 [25]	Case series	7	USA	Male patients ranged in age from 19–39; one patient received Janssen vaccine; one patient received first dose of Pfizer-BioNTech vaccine; four patients received second dose of Pfizer-BioNTech vaccine; one patient received first dose of Moderna vaccine. Myocarditis diagnosis based on consistent clinical symptoms, elevated troponin levels, echocardiogram, and CMR imaging results.	All patients received therapeutic interventions (including beta blockers, steroids, colchicine, etc.) and remained in the hospital for 2–4 days. Symptoms resolved in all patients by the time of discharge.	Further studies are needed to determine if the incidence rate of myocarditis development is higher in the post-vaccinated population compared to the background population. COVID-19 vaccines are still favorable for patients to receive given the risk-benefit of COVID-19 infection.
Vidula et al., 2021 [26]	Case series	5	USA	Three females and two males developed heart-related complications (two myocarditis, two pericarditis, and one cardiomyopathy) within days after vaccination (three patients received second dose of Pfizer-BioNTech vaccine; one patient received second dose of Moderna vaccine; one patient received first dose of Pfizer-BioNTech vaccine).	All patients received therapeutic interventions and were discharged.	A causal relationship between mRNA vaccines and myocarditis development cannot be completely proven.
Singh et al., 2021 [27]	Case report	1	USA	A 24-year-old male received second dose of Pfizer-BioNTech vaccine; time to presentation was 72 h post-vaccination. A cardiac MRI confirmed the diagnosis of myocarditis.	Patient was hospitalized for 4 days and discharged under stable condition. Follow up 6 weeks later showed no further complications.	Other causes of myocarditis were excluded through an extensive work-up. No causal relationship between myocarditis and mRNA vaccine can be developed at this time.
McLean and Johnson, 2021 [28]	Case report	1	USA	A 16-year-old male received second dose of Pfizer-BioNTech vaccine; time to presentation was 60 h post-vaccination. Patient diagnosed with suspected myopericarditis based on clinical presentation and laboratory findings.	With therapeutic intervention of 4-dose immunoglobulins, an ECG showed resolution of ST elevation on hospital day 3. Patient was discharged by day 6 on a seven-day course of ibuprofen and famotidine for symptomatic management.	Cause of myocarditis in this patient was not identified; only the temporal association between COVID-19 vaccination and myocarditis development was established.
Muthukumar et al., 2021 [29]	Case report	1	USA	A 52-year-old male received second dose of Moderna vaccine; time to presentation was 72 h post-vaccination. Diagnosed myocarditis was confirmed based on 2018 Lake Louise criteria.	Symptoms were relieved 3 h after onset. Patient was discharged symptom-free after 4 days with no recurrence >3 months after.	Although this case does not prove a temporal relationship between myocarditis and mRNA vaccines, all other potential causes of myocarditis (such as cardiac injury and viral exposure) were excluded. Providers should remain watchful for the development of myocarditis symptoms following mRNA vaccination.
Abu Mouch et al., 2021 [30]	Case series	6	Israel	Male patients (median age of 22) who developed myocarditis after the second (five patients, within 3 days post-vaccination) or first (one patient, after 16 days) dose of the Pfizer-BioNTech vaccine; time to myocarditis presentation was 24–384 h post-vaccination. Myocarditis was confirmed based on cardiac MRI.	Disease course was mild, leading to discharge 4–8 days after admission.	This report on the occurrence of myocarditis in patients after vaccination could potentially be considered as an adverse reaction to the immunization.
Park et al., 2022 [31]	Case series	2	USA	Males aged 15 and 16 years; one patient received first dose of Pfizer-BioNTech vaccine; the other patient received second dose of Pfizer-BioNTech vaccine; time to presentation was 48–72 h post-vaccination. Myocarditis was confirmed based on elevated troponin levels, ST segment elevation, and cardiac MRI results.	Symptoms were self-limiting and both were discharged after 4 days of admission.	No other etiology of myocarditis was found in these patients. The temporal relationship between the mRNA vaccine and myocarditis development raises the possibility of a “vaccine-related self-limited myocarditis”.
Larson et al., 2021 [32]	Case series	8	USA, Italy	Male patients ranging in age from 21–56; three patients received second dose of Moderna vaccine; four patients received second dose of Pfizer-BioNTech vaccine; one patient received first dose of Pfizer-BioNTech vaccine. Time to presentation was 48–96 h post-vaccination. Patients were diagnosed with myocarditis based on laboratory and cardiac MRI findings.	All were non-fatal, with symptom resolution and discharge.	Current research shows the development of post-vaccination myocarditis to be rare. Providers should remain cautious of myocarditis symptoms following vaccination, but further research is needed to fully determine whether there is a causal relationship.
Kim et al., 2021 [33]	Case series	4	USA	Patients (three males and one female) ranged in age from 23–70; two received second dose of Moderna vaccine; two received second dose of Pfizer-BioNTech vaccine; time to presentation was 24–120 h post-vaccination. Myocarditis diagnosis based on elevated troponin levels, CMR imaging abnormalities, and symptom presentation.	Patients received conservative therapeutic interventions in the hospital and were discharged within 2–4 days.	Diagnosis of acute myocarditis in the patients of this case series was straightforward. It is possible that the cases represent a rare adverse effect of a mRNA vaccine.
Bautista García et al., 2021 [34]	Case report	1	Spain	A 39-year-old male received second dose of Pfizer-BioNTech vaccine; time to presentation was 6 h post-vaccination. Cardiac MRI was performed, showing features compatible with acute myocarditis. Other possible cardiac events, such as coronary disease, were ruled out with testing, leading to a diagnosis of acute myocarditis.	Post-treatment clinical outcome was good; patient was discharged symptom-free after 6 days.	The temporal association between onset of myocarditis symptoms and vaccination, combined with the exclusion of other cardiological etiologies, suggested that the development of myocarditis was an adverse effect to the BNT162b2 vaccine. It is further suggested that this adverse effect could be more predominant in genetically predisposed individuals, as well as those with prior health conditions.
Shaw et al., 2021 [35]	Case series	4	USA	Acute myocarditis developed in four patients (two females and two males; median age 20 years) within days of receiving the mRNA vaccines (three Pfizer-BioNTech, one Moderna) occurring post-second dose in two patients (no prior COVID-19 infection) and post-first dose in two patients with prior COVID-19 infection. Time to presentation was 48–600 h post-vaccination. All findings from patient workups were consistent with 2018 Lake Louise criteria for myocarditis.	Treatment details not provided.	Although the potential temporal association between onset of myocarditis and vaccination was possible in this case series, it does not directly prove that vaccination caused the onset. The possibility of spontaneously occurring myocarditis cannot be excluded.
Mansour et al., 2021 [36]	Case series	2	USA	One male and one female aged 25 and 21 years, respectively; both patients received second dose of Moderna vaccine; time to presentation was 24–48 h post-vaccination. Myocarditis diagnosis was confirmed using 2018 Lake Louise criteria.	Both patients were treated, with symptom resolution and discharged within 3 days of admission.	A relationship between the onset of myocarditis symptoms and vaccination is purely speculation in this case. Further data are needed to determine the exact relationship between myocarditis development and vaccination.
d’Angelo et al., 2021 [37]	Case report	1	Italy	A 30-year-old male received second dose of Pfizer-BioNTech vaccine; time to presentation was 72 h post-vaccination. Laboratory data of elevated cardiac and inflammatory enzymes, along with cardiac MRI, were used to confirm myocarditis diagnosis.	Case was non-fatal; patient was discharged 7 days after admission.	Authors speculated that the development of myocarditis is possibly due to recent immunization with the mRNA vaccine based on the temporal relationship between the two events.
Starekova et al., 2021 [38]	Retrospective study	5	USA	Retrospective identification of four males and one female ranging in age from 17–38 years who were diagnosed with myocarditis/pericarditis within 3 days of receiving the second dose of the mRNA vaccine (three Pfizer-BioNTech, two Moderna). Confirmed diagnosis of myocarditis was based on 2018 updated Lake Louise criteria.	Treatment details not provided.	The clinical presentation and temporal relationship between onset of symptoms and time from vaccination suggests a possibility of vaccine-related myocarditis. The observations in this case series are not indicative of causation, and further research is needed to determine the true relationship between vaccination and onset to myocarditis symptoms.
Tano et al., 2021 [39]	Case series	8	USA	Male patients ranging in age from 15–17 years; six patients received second dose of Pfizer-BioNTech vaccine; two patients received first dose of Pfizer-BioNTech vaccine; time to presentation was 24–96 h post-vaccination. All eight patients presented with acute onset retrosternal pain and elevated troponin levels. Seven patients had ST-segment changes on ECG. Three patients showed myocardial edema and late gadolinium enhancement with cardiac MRI.	All were discharged symptom-free within 4 days of admission.	All patients in this case study were diagnosed with perimyocarditis with no alternate etiology other than recent BNT162b2 vaccination. Although this case series cannot conclude a causal relationship between vaccination and myocarditis, providers should still be cautious when patients present with myocarditis symptoms post-vaccination.
Watkins et al., 2021 [40]	Case report	1	USA	A 20-year-old male received second dose of Pfizer-BioNTech vaccine; time to presentation was 48 h post-vaccination. Myocarditis diagnosis was confirmed with cardiac MRI.	Patient recovered and was discharged on medications.	Authors suggested that this case showed a direct temporal relationship between onset of myocarditis symptoms and time from mRNA vaccination. Other potential causes of myocarditis were deemed unlikely, but this case report cannot definitely claim a cause-and-effect relationship between myocarditis and mRNA vaccination.
Williams et al., 2021 [41]	Case report	1	Canada	A 34-year-old male received second dose of Moderna vaccine; time to presentation was 72 h post-vaccination. Laboratory testing and cardiac imaging were performed, which confirmed diagnosis of myocarditis per Lake Louise criteria.	Discharged without symptoms 7 days after admission.	This case report states that the purpose of the case was to bring light to a rare, but potential adverse effect of vaccinations.
Habib et al., 2021 [42]	Case report	1	Qatar	Case of a 37-year-old male who developed myocarditis 3 days after receiving the second dose of the Pfizer-BioNTech vaccine. Diagnosis of myocarditis was confirmed by CMR imaging.	Discharged 6 days after admission.	The most likely diagnosis in this patient is vaccine-related myocarditis. Conclusion was based on the acute onset, temporal association, and exclusion of alternate etiologies in this patient. Further research is needed to determine the relationship between myocarditis onset and vaccination.
Cereda et al., 2021 [43]	Case report	1	Italy	A 21-year-old male received second dose of Pfizer-BioNTech vaccine; time to presentation was 30 h post-vaccination. Cardiac MRI confirmed the myocarditis diagnosis.	Discharged symptom-free after a week of admission.	The development of myocarditis after vaccination can either be causally related or by chance. There are two possible hypotheses for myocarditis development in this patient. The vaccine could have triggered an autoimmune response that resulted in myocarditis, or the vaccine caused an autoimmune/inflammatory response that progressed to myocarditis. Risk-benefit decision is still in favor of patients receiving the vaccine, so providers should just be cautious.
Hudson et al., 2021 [44]	Case series	2	USA	Cases of two male patients (22- and 24-year-old) who developed myocarditis within 3 days of receiving the second dose of the Pfizer-BioNTech vaccines. ECG findings, symptoms, and/or laboratory testing were used to give a diagnosis of probable myocarditis.	Patients were discharged within 3 days of hospital admission.	Further data are needed to determine a relationship between myocarditis development and vaccination, but providers should remain cautious of patients who present with symptoms of myocarditis after recent vaccination.
Isaak et al., 2021 [45]	Case report	1	Germany	A 15-year-old male received second dose of Pfizer-BioNTech vaccine; time to presentation was 24 h post-vaccination. Diagnosis of myocarditis was confirmed per fulfillment of 2018 Lake Louise criteria.	Discharged within 7 days of hospitalization and treatment.	Providers should be made aware of the possibility of vaccine-related myocarditis.
Snapiri et al., 2021 [46]	Case series	7	Israel	Male patients aged 16 or 17; six patients received second dose of Pfizer-BioNTech vaccine; one received first dose of Pfizer-BioNTech vaccine; time to presentation was 24–72 h post-vaccination. Diagnosis of perimyocarditis was made based on suitable clinical presentation of elevated cardiac biomarkers, abnormal echocardiogram, or ECG findings.	All cases were non-fatal with patients discharged symptom-free within 6 days of hospitalization.	COVID-19 could be responsible for the development of myocarditis in this case series. There were differential diagnoses and pathogenesis of the patients, and COVID-19 itself cannot be ruled out as a possible cause. Further research is needed to determine if vaccine-related myocarditis is an adverse effect of an mRNA vaccine.
Hasnie et al., 2021 [47]	Case report	1	USA	A 22-year-old male received first dose of Moderna vaccine; time to presentation was 72 h post-vaccination. CMR imaging was consistent with diagnosis of perimyocarditis.	Symptoms resolved after treatment and patient was discharged, with joint decision to forgo the second dose.	A temporal relationship exists between the development of perimyocarditis and recent COVID-19 vaccination in this patient. Case report highlights a rare adverse effect of COVID-19 vaccinations. Further research is needed to determine the true incidence of this adverse effect in the population.
Tailor et al., 2021 [48]	Case report	1	USA	A 44-year-old male received second dose of Moderna vaccine; time to presentation was 96 h post-vaccination. Cardiac MRI confirmed diagnosis of acute myocarditis.	Supportive treatment and 1 month follow-up confirmed complete symptom resolution.	The patient in this case report had typical clinical and laboratory findings consistent with myocarditis and was diagnosed with vaccine-related myocarditis due to the strong temporal relationship of his symptom development following vaccination. This adverse effect is expected to be rare, and this case report does not prove a temporal relationship.
Patel et al., 2021 [49]	Case series	5	USA	Male patients ranging in age from 19–37; one patient received first dose of Pfizer-BioNTech vaccine; three patients received second dose of Pfizer-BioNTech vaccine; one patient received second dose of Moderna vaccine; time to presentation was 24–72 h post-vaccination. CMR was used to confirm diagnosis of acute myocarditis.	Three patients were treated with colchicine with or without aspirin/ibuprofen. Two patients were not medically treated. All patients were discharged in stable clinical condition.	This case series adds to the growing number of cases that suggest a relationship between mRNA vaccines and myocarditis development. Further research is needed to make a definitive conclusion about the possibility of vaccine-related myocarditis as an adverse effect of mRNA COVID-19 vaccines.
Mevorach et al., 2021 [51]	Retrospective study	283	Israel	Forty-eight percent of myocarditis cases were definitive or probable after receipt of Pfizer-BioNTech mRNA vaccine. Overall risk difference between first and second dose was 1.76 per 100,000 persons. Standardized incidence ratio was 5.34.	Overall, clinical presentation was mild, but one patient experienced fulminant myocarditis that was fatal.	Incidence of myocarditis occurred predominantly in males under the age of 30. Myocarditis after second Pfizer BioNTech dose had the highest standardized incidence ratio for male recipients between the ages of 16 and 19 years (13.60 per 100,000 doses).
Witberg et al., 2021 [52]	Retrospective cohort study	54	Israel	Highest incidence of myocarditis occurred among young male patients between ages of 16 and 29 that had received at least one dose of Pfizer-BioNTech (10.69 per 100,000).	Seventy-six percent of cases were mild, 22% intermediate, and one case was associated with cardiogenic shock. One patient with preexisting cardiac disease died of an unknown cause soon after hospital discharge.	Clinical presentation of myocarditis was generally mild and seen most in males under the age of 30.
Barda et al., 2021 [53]	Retrospective observational cohort study	884,828	Israel	Vaccination against COVID-19 with Pfizer-BioNTech was most strongly associated with an increased risk of myocarditis, with a risk ratio of 3.24.	Not indicated	COVID-19 vaccination was associated with an excess risk of myocarditis. The risk of this adverse event, along with others, was also substantially increased after SARS-CoV-2 infection. The Pfizer BioNTech vaccine was associated with an increased risk of adverse events over a 42 day follow up period, but COVID-19 infection itself could also increase the risk of adverse events, potentially ones that are much more probable to be fatal.
**Secondary Immune Thrombocytopenia**						
Tarawneh and Tarawneh, 2021 [54]	Case report	1	USA	Case of 22-year-old male who developed petechiae and gum bleeding 3 days after receiving the first dose of the Pfizer-BioNTech vaccine. Laboratory tests confirmed severe thrombocytopenia.	Patient received platelet transfusion and intravenous immunoglobulin. Symptoms improved and was discharged 3 days after emergency hospitalization.	There is no certainty that this case of thrombocytopenia was vaccine-induced and may have been coincidental. Another consideration is that 2 months prior, the patient’s platelet count was near the lower limit of normal, so it is difficult to exclude other causes.
Lee et al., 2021 [55]	Case series	20	USA	Reports, identified through registry/databases, described cases of 20 patients with varying levels of secondary thrombocytopenia following vaccination with Moderna or Pfizer-BioNTech vaccines	Cases were nonfatal.	Authors recommend getting a baseline platelet count in patients with pre-existing thrombocytopenia before receiving either the Pfizer-BioNTech or Moderna vaccine and a follow-up platelet count after vaccination. Study also showed possible treatment options for patients with potential secondary ITP; most patients responded favorably to treatment with corticosteroids or IVIG, which points to an immune-mediated mechanism that characterizes ITP.
Welsh et al., 2021 [56]	Case series	28	USA	Analysis of 28 VEARS-reported cases of thrombocytopenia (15 cases after the Pfizer-BioNTech vaccine and 13 cases after the Moderna vaccine).	Not indicated	Incidence considered rare based on the reporting rate. Many patients who were affected had comorbidities, including autoimmune disorders or prior history of ITP. Authors concluded that there is no safety concern associated with the mRNA COVID-19 vaccines at this time.
Helms et al., 2021 [57]	Case report	1	USA	Case of a 74-year-old male who developed refractory thrombocytopenia within 24 h post-vaccination with the Moderna vaccine.	Patient received platelet transfusion and intravenous immunoglobulins. Patient improved and was transferred to skilled nursing facility 25 days post-vaccination.	The authors stated that it is uncertain whether refractory ITP secondary to vaccination with a mRNA COVID-19 vaccine will be a rare adverse event. Nonetheless, this uncertainty should not limit the use of mRNA vaccination as a public health measure to prevent or mitigate COVID-19 infection.
**Allergic Hypersensitivity Reactions**						
Dages et al., 2021 [58]	Single-center, retrospective cohort study	68	USA	The goal was to determine if patients with a history of atopic disease on subcutaneous immunotherapy (SCIT) are at an increased risk of developing an allergic reaction to the COVID-19 vaccine.	Within the cohort, none of the patients experienced an allergic reaction (including an anaphylactic reaction) to a mRNA COVID-19 vaccine.	It is recommended that patients with a history of allergic reactions have an observation time of 30 min post-vaccination instead of the standard 15 min of observation. Due to the small sample size, it is difficult to recommend the timing of administration between the COVID-19 vaccine and SCIT.
Krantz et al., 2021 [59]	Case series	47	USA, Denmark	Of the total patients referred for potential immediate allergic reactions to the Pfizer- BioNTech mRNA vaccine, 39 had histories of mild reactions and eight had anaphylaxis to the first vaccine dose. The eight patients with anaphylaxis were supervised during receipt of their second dose.	Eight patients with anaphylaxis to the first Pfizer-BioNTech dose tolerated the second dose.	The eight patients who tolerated a second dose despite anaphylaxis to the first suggests a non-IgE-mediated mechanism. The authors state that those with potential for anaphylaxis should undergo risk stratification to weigh the benefit and risk of a second dose.
**CNS and Orofacial Events**						
Koh et al., 2021 [60]	Multi-center, prospective observational cohort study	457	Singapore	A total of 457 individuals out of about 1.4 million vaccinated persons developed neurological disorders.	Acute ischemic stroke was the most common cerebrovascular disorder (*n* = 243). The most common post-vaccination CNS syndrome was seizures (*n* = 33). Eleven patients experienced Bell’s Palsy after vaccination with the Pfizer vaccine. One patient who experienced cerebral venous thrombosis remained neurologically debilitated after discharge for 6 weeks. Otherwise, patients had good functional outcomes upon discharge.	No neurological morbidity was found. Authors concluded that their observational study does not establish causal relationships between the reported neurological complications and recent mRNA vaccination. The benefits of COVID-19 mRNA vaccination exceed any potential concerns for neurological adverse effects.
García-Grimshaw et al., 2021 [61]	Prospective observational cohort study	4258	Mexico	Observational study of 704,003 first-doses recipients of the Pfizer-BioNTech vaccines showing an observed frequency of serious neurological adverse events at 47/million doses.	Non-serious neurological adverse events post-vaccination had an overall incidence rate of 600.7 cases per 100,000 administered doses. Headache was the most frequent (62.2%). Serious neurological adverse events post-vaccination had an overall incidence rate of 2.4 cases per 100,000. 76.3% of neurological adverse events reported in this study were female.	Pfizer-BioNTech mRNA vaccine is predominantly safe and effective. Concerns about potential neurological adverse effects should not limit the use of vaccination to reduce COVID-19 severity and mortality.
Saito et al., 2021 [62]	Case report	1	Japan	Case report of 42-year-old female who developed aseptic meningitis one week after receiving the first dose of the Pfizer-BioNTech vaccine.	Five days after admission, headache and nausea were significantly improved. Patient was discharged at day 7 post-admission, and experienced no headache, nausea, or fever for at least 4 months after discharge.	Authors concluded that results suggested a rare type of immune-mediated aseptic meningitis, with molecular mimicry as a potential mechanism.
Eom et al., 2022 [63]	Case series	2	Republic of Korea	Case series of male (81 years of age) and female (23 years of age) who developed acute transverse myelitis 3 days after the second dose, and 3 weeks after the first dose, respectively. Both received the Pfizer-BioNTech vaccine.	One month after the end of treatment, male patient still had limitation of finger movements. Three weeks after treatment, leg weakness in female patient improved and she could walk with unilateral assistance.	Causation could not be established between the patients’ neurological symptoms and mRNA COVID-19 vaccination.
Chun et al., 2021 [64]	Case series	9	Republic of Korea	Patients (median age of 7) who developed various mild oral symptoms following a median of 3 days post-vaccination (four Pfizer-BioNTech; five Astra Zeneca).	All the patients’ symptoms responded to treatment within a short time.	This case series suggests oral mucositis, ulceration, and neuropathic pain as possible orofacial adverse effects following COVID-19 vaccination. Clinicians should understand the potential risk and guide patients appropriately to lower vaccine hesitancy.
Mazur et al., 2021 [65]	Survey-based study	223	Italy, Poland, France, Germany, Switzerland, Albania, Denmark, Norway, UK, Spain	Survey of 700 individuals who received COVID-19 vaccines with 3.1% and 5.4% reportedly experiencing oral and facial symptoms, respectively.	Among oral symptoms, burning was the most common adverse effect, with 2.7% and 3.4% after the first and second dose, respectively.	This study observed no significant correlation between vaccine administration for COVID-19 and orofacial manifestations.
**Dermatological Reactions**						
McMahon et al., 2021 [66]	Provider-facing registry-based study	414	USA	For the period analyzed, study identified 414 cutaneous reactions to mRNA COVID-19 vaccines from Moderna (83%) and Pfizer-BioNTech (17%) vaccination.	No serious adverse events developed in any registry patients following either the first or second dose of mRNA vaccine.	Reported cases were predominantly female (90%), white (78%), and from the US (98%). Cutaneous side effects of mRNA vaccination are usually self-limiting, without requiring any therapeutic intervention. The risk of cutaneous adverse events does not outweigh the benefits of COVID-19 vaccination.
Edriss et al., 2021 [67]	Case report	1	USA	Case of 54-year-old male who developed a pruritic rash one week after receiving the first dose of the Pfizer-BioNTech vaccine in the same arm.	Treatment yielded improvement at the first follow-up visit.	Cutaneous reactions after receipt of the Pfizer-BioNTech mRNA vaccine are treatable; therefore, the benefit of receiving COVID-19 vaccination outweighs the risks.
Cyrenne et al., 2021 [68]	Case series	2	Canada	Two cases of suspected vaccine-associated skin reactions, first in a woman in her 20s two days after her first-dose vaccination and the second in a male in his 40s three weeks after the second dose. Both received the Pfizer-BioNTech mRNA vaccine.	One female patient was treated with topical corticosteroids, and it resolved her diagnosis of a pityriasis rosea-like eruption. One male patient was given doxycycline and bilastine to resolve his pityriasis-rosea-like eruption.	Further research is needed to determine if causality exists between mRNA COVID-19 vaccination and dermatological adverse events, but providers should be aware of the various cutaneous manifestations reported. Authors recommended that clinicians consider providing pre-vaccination counseling on topical medication use and symptom alleviation.

Abbreviations: CMR—cardiac magnetic resonance; ECG—electrocardiogram; LVEF—left ventricular ejection fraction; MRI—magnetic resonance imaging; VAERS—Vaccine Adverse Event Reporting System.

## Data Availability

Dataset openly available in the public domain.
